# The Challenges Faced and Lessons Learnt in the Multidisciplinary Management of Medication Refractory Post-Stroke Sialorrhea: A Case Report

**DOI:** 10.7759/cureus.7978

**Published:** 2020-05-05

**Authors:** Siew Kwaon Lui, Shirlyn Hui Shan Neo, Daniel Song Chiek Quah

**Affiliations:** 1 Rehabilitation Medicine, Singapore General Hospital, Singapore, SGP; 2 Supportive and Palliative Care, National Cancer Centre, Singapore, SGP; 3 Radiation Oncology, National Cancer Centre, Singapore, SGP

**Keywords:** sialorrhea, salivary gland, radiotherapy, radiation therapy, stroke, case report

## Abstract

Post-stroke sialorrhea is a very debilitating condition that negatively impacts a person’s physical and psychological health. In this report, we discuss a case of medication refractory post-stroke sialorrhea in a female patient. The patient received multidisciplinary management and two fractions of radiotherapy over a course of five months. The treatment was successful, and the patient reported satisfactory control of her sialorrhea symptoms. We hope to highlight the importance of multidisciplinary management and consideration of radiotherapy as a treatment modality when providing care for medication refractory post-stroke sialorrhea patients who are unsuitable for salivary gland botulinum toxin (BoNT) injection and surgery.

## Introduction

Sialorrhea is defined as the involuntary flow of saliva beyond the lip margin [1]. The most common cause of post-stroke sialorrhea is related to the neurological impairment that involves either an inability to retain saliva within the mouth secondary to poor orofacial muscular control and/or dysphagia leading to excess pooling of saliva in the anterior oral cavity [2,3]. Sialorrhea can lead to perioral infections, aspiration pneumonia, impaired communication, as well as negative effects on the mental and psychological well-being of both the patient and caregiver [4]. Sialorrhea is often undertreated [5]. Hence, rehabilitation medicine physicians who are diagnosing and treating disabling conditions in stroke patients need to diagnose and treat sialorrhea in the post-stroke cohort as well as work collaboratively with other specialists to devise effective sialorrhea treatment with minimal side-effects. We report a case of post-stroke sialorrhea, the management of which was challenging. It required multidisciplinary management as well as the consideration of radiotherapy as a treatment modality.

## Case presentation

A 67-year-old female with a history of bilateral middle cerebral artery infarcts and atrial fibrillation (AF) presented with a four-year history of sialorrhea that had severely affected her quality of life (QOL). She spat every two to three hours, packed tissues into her mouth to dry up her saliva, and required 10 changes of tissues per day. She had been regularly reviewed by a speech therapist and had been found to have moderate to severe oropharyngeal dysphagia, characterized by limited and effortful bolus manipulation propulsion, delayed swallow triggers, reduced hyolaryngeal elevation, and reduced epiglottic retroflexion. She also had severe dysarthria characterized by limited articulation, reduced voicing, impaired resonance, and poor respiratory support. She received oral motor therapy, which included the jaw, tongue, and tongue-to-palate exercises.

Her pharmacological treatment for sialorrhea included oral hyoscine butylbromide, which provided limited relief, and a scopolamine patch, which resulted in adverse effects of drowsiness and confusion. As glycopyrrolate was only available in injectable form in our local hospital, and the option of her carer syringing the glycopyrrolate from the vial was not feasible, oral glycopyrrolate could not be prepared using the injectable glycopyrrolate by the dilutional method. Salivary gland injection with botulinum toxin (BoNT) was considered. However, as her international normalized ratio (INR) levels often exceeded 3.0 related to anticoagulation, the risks of bleeding with BoNT salivary gland injection outweighed the benefits, and this option was not further pursued. 

She was referred to the palliative medicine specialist for further management. Her antidepressant medication, fluoxetine, was switched to nortriptyline, which was gradually increased from 10 mg nightly to 100 mg nightly. As she had a history of AF and constipation, the nortriptyline dosage could not be further increased. The addition of atropine drops to the pharmacological regime did not provide any additional symptomatic relief. As her sialorrhea was still troubling her, she was subsequently referred to the radiation specialist for consideration of radiotherapy for sialorrhea management.

The risks and benefits of radiotherapy were discussed with her, including the small risk of radiation-induced malignancies. She consented and was given one fraction of radiotherapy (7.5 Gy) using photons delivered by parallel-opposed lateral fields. She reported transient sore throat after radiotherapy. After three months, she reported significant improvement of her sialorrhea (her use of eight gauzes a day came down to two gauzes a day). Nortriptyline dose was reduced to 25 mg nightly. Two months later, she reported gradual recurrence of her sialorrhea (her use of five gauzes a day had increased to eight gauzes a day). She was given a second fraction of radiotherapy (7.5 Gy), this time using electrons delivered in left and right lateral fields. One month later, she reported satisfactory relief of her sialorrhea with a reduction of usage of gauzes to three to four a day. Nortriptyline was weaned off. Two months after her second fraction of radiotherapy, her Drooling Rating Scale (DRS) score markedly decreased from 15 (constant use of cotton gauze during sitting, standing, in bed, talking, eating, and drinking) to zero (excessive dryness of the mouth). At the four-month post-radiotherapy follow-up, she reported using three gauzes a day and she was quite happy with her symptom improvement. She has continued with her three-month follow-up visits with the radiation specialist and her six-month follow-up with the rehabilitation medicine physician.

## Discussion

Adult sialorrhea is most commonly studied in Parkinson’s disease (PD) due to its high prevalence (70%) [3]. Hence, most of the literature on the management of sialorrhea deal with PD cases. Although the prevalence of sialorrhea in stroke has not been well-documented, it can lead to medical, psychological, and social complications in stroke patients [4]. The key to the treatment of sialorrhea is multidisciplinary management by a team of specialists that would ideally include a rehabilitation medicine physician, speech therapist, occupational therapist, physiotherapist, otolaryngologist, neurologist, palliative medicine specialist, and radiation specialist [1,3,4].

The assessment of sialorrhea is mainly based on a clinical diagnosis, which includes the use of objective and subjective measures with the aim to quantify the condition [6]. Objective measures include measuring the volume of saliva, observing the number of drooling episodes in a fixed duration of time, and weighing cotton gauzes used to contain the saliva production; however, these measures are impractical and often inaccurate due to inefficient collection methods [7]. Three common subjective measures used to evaluate sialorrhea are the Drooling Severity and Frequency Scale (DSFS), Drooling Rating Scale (DRS), and the Sialorrhea Clinical Scale for PD (SCS-PD) [8]. The DSFS consists of a 5-point rating scale grading drooling severity and a 4-point rating scale grading drooling frequency [8]. The DRS assesses the severity of drooling in several situations such as during sitting, standing, staying in bed, talking, eating, or drinking based on a 0-3 point rating scale [8]. Both DSFS and DRS do not assess the psychosocial effects of sialorrhea [8]. The SCS-PD consists of a 0-3 point rating scale of seven questions covering the severity, frequency, and feeling of discomfort during day-time, night-time, eating, speaking, and social participation [8]. Table 1 shows a comparison of the three scales.

**Table 1:** A comparison of DSFS, DRS, and SCS-PD scales DSFS: Drooling Severity and Frequency Scale; DRS: Drooling Rating Scale; SCS-PD: Sialorrhea Clinical Scale for Parkinson’s Disease

DSFS	DRS	SCS-PD
Measures the severity and frequency of sialorrhea symptoms	Measures the severity of sialorrhea symptoms only	Measures the severity and frequency of diurnal and nocturnal sialorrhea symptoms
Does not assess functional impairment	Does not assess functional impairment	Assesses functional impairment of speech and eating
Does not assess social impairment	Does not assess social impairment	Assesses social impairment, e.g., whether bothered by other people noticing the sialorrhea symptoms or whether stopped social interaction because of sialorrhea symptoms
Has been validated in children with a variety of neuromuscular disorders including cerebral palsy, intellectual disability, and developmental delay, showing correlation with the objective measure, the Drooling Quotient, which quantifies the number of drooling episodes occurring over two observation sessions, e.g., counting the number of occasions when drool was present or absent, measured at 15-second intervals over a 10-minute period [9]	Has not been validated	Has been validated in PD patients and controls, showing correlations with objective saliva volume measurements [10]

Currently, there is no consensus as to which subjective scale is the standardized measure, and none of them have been validated in post-stroke patients [1]. As the treatment of sialorrhea aims to improve QOL of the affected patients and their families, a 1990 consortium on the management of sialorrhea concluded that objective quantification of sialorrhea was not required to determine treatment effectiveness [1]. For our patient, we assessed the symptom control by reviewing her history of daily use of the number of cotton gauzes and assessing her DRS score. The DRS measure was used due to its ease of administration although we acknowledged its pitfalls related to inadequate assessment of social and functional impairment.

Non-pharmacological treatment includes behavioral modification, use of oral appliances, and multidisciplinary rehabilitation using occupational, physical, and speech therapy to improve head control and muscle tone [1]. Pharmacological treatment consists primarily of anticholinergic medications, which include atropine, scopolamine (hyoscine), glycopyrrolate, and a tricyclic antidepressant (amitriptyline, nortriptyline) and/or BoNT salivary gland injection. Anticholinergic medications usually provide short-term relief and are not as well-tolerated in elderly patients as they are associated with sedation, confusion, irritability, hyperactivity, xerostomia, urinary retention, and constipation; however, glycopyrrolate has a lower risk of central side-effects due to its quaternary ammonium structure, which renders it less likely to cross the blood-brain barrier [1,6]. However, approximately 30-35% of patients still discontinue the use of glycopyrrolate due to its side-effects [6]. As for BoNT salivary gland injection, the reported response rates for sialorrhea treatment range from 40 to 100%, and the duration of effect varies between 2-36 weeks [11-13]. Hence, repeated BoNT injections may be needed for long-term control.

For our patient, we had exhausted all pharmacological treatment options including oral hyoscine butylbromide, atropine, nortriptyline, and topical scopolamine patch. Oral glycopyrrolate was not prescribed as it was unavailable; also, it would require a dedicated and competent carer to prepare the oral form by withdrawing from the glycopyrrolate vial with a needle and syringe and adding to a fixed volume to prepare a 1:1 oral mixture, an option that was deemed not feasible after discussing with the patient. Salivary gland BoNT injection was not pursued due to her fluctuating INR levels, which often exceeded 3.0. To the best of our knowledge, there have been no reports on the safety of salivary gland BoNT injection in patients whose INR levels exceeded 3.0. Safety studies and consensus statements related to BoNT injections in patients with oral anticoagulation are limited to intramuscular BoNT injections in cases of blepharospasm, cervical dystonia, and spasticity management, where the INR cut-off levels are either <3.0 or <3.5 [14,15].

Options such as surgery and radiotherapy are considered in sialorrhea management when other approaches have been exhausted [3]. Surgery generally involves a combination of gland removal, duct ligation, or duct relocation of submandibular glands or/and parotid glands [1]. Surgical complications may include facial nerve palsy, oedema, pain, paraesthesia, and hemorrhage [1,2]. Many neurological patients, especially older ones and those with multiple co-morbidities, may be medically unfit to undergo surgery. The surgical risks may also outweigh the benefits in neurological patients with a limited life expectancy, such as those with amyotrophic lateral sclerosis [16]. Our patient, who was aged more than 65 years, with a history of AF, bilateral strokes, and receiving anticoagulation, arguably had a higher risk for surgery and possibly a shorter life expectancy. Moreover, all pharmacological treatment options had been exhausted in her case. Radiotherapy was therefore considered for her treatment.

Radiotherapy is the use of ionizing radiation, usually in the treatment of malignancies. When used in head and neck malignancies, one of the common side-effects is decreased saliva production and, consequently, xerostomia. Radiation works by causing acinar cell atrophy and fibrosis, changes in vascular connective tissue, and neurologic function [17]. Serous acini are usually affected before mucous acini, thereby leading to thick viscous secretion. There have been suggestions that the submandibular gland is more affected than parotid glands by radiotherapy. The effects of xerostomia may last up to six months and may be permanent in some cases. This has led to the consideration of using radiotherapy in the treatment of sialorrhea that is medication refractory or unsuitable for more common interventions.

Common adverse effects of radiotherapy to salivary glands include pain, loss of taste, xerostomia, and viscous saliva; however, these effects are mostly transient [18]. The risk of malignancy after radiation is low (one to three per 100,000 people per year), and the reported latency period ranges from six to 32 years [19]. The use of radiation for sialorrhea management is not advised in younger patients whose lifespan is predicted to be prolonged (in the order of decades) due to risks of inducing malignancy and delayed growth, which can lead to facial asymmetry, mucositis, xerostomia, dental decay, and osteoradionecrosis [1]. There is currently no consensus about the type of radiation and optimal dosing regimen for salivary gland irradiation [20]. However, in light of the poor functional status of our patient and the difficulty she faces in traveling to and from the hospital, a single fraction of radiotherapy was used to minimize inconvenience. The most commonly used regimens target both submandibular glands and the caudal two-thirds of both parotid glands [20]. There is evidence that QOL improves significantly after radiotherapy in patients with sialorrhea [18]. The Royal College of Radiologists recommends radiotherapy as an effective treatment tool in palliating sialorrhea in patients with advanced neurodegenerative disorders [19].

## Conclusions

Sialorrhea is a vexing problem and can severely affect the QOL in post-stroke patients. The challenging case of medication refractory post-stroke sialorrhea that we reported highlights the importance of multidisciplinary management in sialorrhea treatment. A step-up approach starting with the least destructive and non-invasive treatment is generally advocated in sialorrhea management. Non-invasive approaches that are generally reserved as last resort, such as radiotherapy, can be considered in post-stroke patients with debilitating sialorrhea who have exhausted pharmacological treatment options and are unfit to undergo surgery. This case illustrates that radiotherapy in sialorrhea treatment improves the QOL, especially in patients who are poor surgical candidates. As with all radiotherapy in general, considerable care should be applied in choosing the right fractionation of radiotherapy that is most appropriate for such frail patients.
